# Elevated Inflammation and Poor Diet Quality Associated with Lower eGFR in United States Adults: An NHANES 2015–2018 Analysis

**DOI:** 10.3390/nu16040528

**Published:** 2024-02-14

**Authors:** Aljazi Bin Zarah, Jeanette Mary Andrade

**Affiliations:** 1 Food Science and Human Nutrition Department, University of Florida, Gainesville, FL 32611, USA; aljazibinzarah@ufl.edu; 2 Community Health Sciences Department, College of Applied Medical Sciences, King Saud University, P.O. Box 10219, Riyadh 11433, Saudi Arabia

**Keywords:** chronic kidney disease, inflammation, diet quality, NHANES

## Abstract

Chronic kidney disease is prevalent within the United States likely due to dietary habits. The purpose of this study was to examine the relationship between the high-sensitivity c-reactive protein (hs-CRP) and diet quality (DQ) and their effect on the eGFR. A cross-sectional secondary data analysis study was conducted among adults (*n* = 6230) using NHANES 2015–2018 data. DQ was determined by the Healthy Eating Index-2015 (HEI-2015). Multivariable linear regressions were conducted based on eGFR (≥90 or <60 mL/min/1.73 m^2^) after adjustments for age, race/ethnicity, hypertension, diabetes mellitus, cardiovascular disease, and kidney disease awareness. All analyses were performed in SAS version 9.4 with a statistical significance of *p* < 0.05. Results showed that participants who had an eGFR of <60 mL/min/1.73 m^2^ were older and had a higher prevalence of hypertension and diabetes and had higher hs-CRP compared to participants with an eGFR ≥ 90 (*p* < 0.005). Of participants with an eGFR < 60, 27% reported that they were aware they had kidney disease. Regardless of the eGFR at baseline, there was a negative interaction effect on the DQ scores and hs-CRP on the eGFR (*p* < 0.05). Independently, for participants with an eGFR < 60, their DQ scores had a positive significant relationship on their eGFR (*p* = 0.03), whereas their hs-CRP had a negative significant relationship on thier eGFR (*p* < 0.001). For participants with an eGFR < 60, age, hypertension, and kidney disease awareness influenced this relationship (*p* < 0.001). Overall, low DQ and elevated hs-CRP contributed to a reduction in kidney function. Efforts to improve dietary intake and strategies to reduce inflammation and improve kidney function are necessary.

## 1. Introduction

Chronic kidney disease (CKD) affects at least 14% of US adults, of which 9 out of 10 do not know they have this disease and 1 out of 3 do not know they are in the later stages of this disease [1]. The stage of CKD is normally determined through the estimated glomerular filtration rate (eGFR) that considers serum creatinine, age, and sex [2]. The lower the eGFR, the higher the severity of the CKD, in that an eGFR of 15 mL/min/1.73 m^2^ would indicate stage five and an eGFR of 60 mL/min/1.73 m^2^ would indicate stage two [2]. A multitude of factors contribute to the progression of CKD such as co-morbidities like diabetes and hypertension, genetics, age, and inflammation.

Persistent low-grade inflammation is a key element of CKD that affects the progression and complications associated with this disease [3]. The two dominant inflammation markers in adults with CKD are interleukin-6 (IL-6) and the C-reactive protein (CRP) [4,5]. IL-6 is a multifunctional cytokine that contributes to immunity because of its broad spectrum of immunological and hematological activities and its intense ability to produce acute phase responses such as CRP [6]. CRP is an acute phase protein synthesized in the liver that has been related to a higher risk of cardiovascular disease (CVD) morbidity and mortality in adults with CKD [4]. CRP has a 19-h half-life, which makes it more capable of detecting inflammatory processes [7]. The levels of CRP are inversely correlated with the degree of inflammation within the body, in which levels could increase after inflammatory stimulation in six hours. Elevated inflammation may be attributed to malnutrition, endothelial dysfunction, coronary artery calcification, atherosclerosis, mineral and bone disease, and, ultimately, mortality. Some strategies to mitigate this rise in inflammation include medications, lifestyle habits, and diets.

According to the current KDOQI (Kidney Disease Outcomes Quality Initiative) guidelines [2], adults with CKD should consume a dietary pattern that is balanced in plant-based foods such as beans and legumes, whole grains, fruits, and vegetables as a method to reduce inflammation and the progression of this disease [8]. The potential mechanism of action is that as the intake of plant-based foods is increased, and, if possible, is the sole source of energy and protein, the net dietary acid load is lowered and in turn lowers inflammation [9]. Additionally, dietary fiber plays a significant role in improving the nutritional status. As the intake of both soluble and insoluble fibers increases, the gut/microbiota health improves, which reduces inflammation [10,11]. Based on observational studies that focus on the whole diet among adults with CKD, results show that adults with CKD consume a greater portion of red/processed meats and sodium and fewer portions of fruits, vegetables, and whole grains [12,13]. For the few studies that assessed the diet quality (DQ) of adults with CKD, the DQ was low related to the consumption of fruits, vegetables, whole grains, and plant-proteins [14,15]. 

DQ is a broad term that describes how closely an individual’s diet adheres to dietary guidelines [16]. In the US, DQ is determined by the Healthy Eating Index-2015 (HEI-2015) [17]. The HEI-2015 is a valid tool that employs a scoring system to measure how closely a dietary pattern adheres to the dietary guidelines for Americans. A low DQ may progress CKD by increasing the high-sensitivity CRP (hs-CRP) resulting in localized inflammation and vascular proliferation in the peritoneal, cardiac, and vascular tissues [18,19]. However, the relationship between DQ and hs-CRP and their interaction on the eGFR in a general population within the United States who has an eGFR of <60 mL/min/1.73 m^2^ has not been extensively explored. The purposes of this study were (1) to determine the interactive effect of DQ and hs-CRP, (2) to explore the dietary components and their relationship with eGFR, and (3) to examine the demographic and health conditions that have an influencing effect on DQ, hs-CRP, and eGFR. The hypotheses were that (1) there exists an interactive effect in which a low DQ with high amounts of hs-CRP results in a lower eGFR, that (2) higher scores of refined grains and total proteins and lower scores of seafood and plant proteins, total fruits, and total vegetables would lower the eGFR, and that (3) age, race/ethnicity, hypertension, diabetes, and kidney awareness would have an influence on the relationship between DQ and hs-CRP and their effect on the eGFR.

## 2. Materials and Methods

### 2.1. Study Design 

A cross-sectional secondary data analysis based on the National Health and Nutrition Examination Survey (NHANES) from two cycles, 2015–2016 and 2017–2018, was conducted. These two data sets were used due to the similarities in the collection and analysis of hs-CRP and the dietary collection methodology. The NHANES uses a complex sampling structure that provides a classification, clustering, weighting, and oversampling of specific population subgroups (e.g., people 60 years of age and over, African Americans, Asians, and Hispanics), in which participants provide written informed consent that is approved through the NHANES research ethics board. Although this study was a secondary analysis of publicly available NHANES data, all study protocols were granted to be exempt and ethical approval was provided by the University of Florida Institutional Review Board #202100655.

A total of 19,225 individuals were sampled from NHANES data from 2015 to 2018. The inclusion criteria included participants (1) who were 18 years of age or older, (2) had demographic data (gender, age, and race/ethnicity), (3) had serum creatinine and hs-CRP measurements, (4) completed two 24-h dietary recalls, and (5) had self-reported health-related conditions. Participants were excluded if they did not meet the above criteria and identified that they were pregnant or that the pregnancy test was inconclusive. The total sample size analyzed was 6230 (Figure S1). Through a sample size and power analysis, as the primary outcome of the study was the eGFR with a mean and SD values of (101.53 ± 22.58) based on a prep-to-research examination, assuming a two-tailed alpha of 0.05, the study had a statistical power of at least 95% to detect a partial correlation of 0.05.

### 2.2. Data Collection

Data collected included self-reported responses to the following questions: demographics (age, gender, and race/ethnicity) and health-related conditions (hypertension, diabetes mellitus, cardiovascular disease, and kidney disease awareness). Data used included serum creatinine to determine the eGFR and amount of hs-CRP. The eGFR was estimated using the updated chronic kidney disease epidemiology collaboration equation (CKD-EPI) and was reported in mL/min/1.73 m^2^ [20]. 

### 2.3. Dietary Assessment 

Two 24-h dietary recalls were collected using the United States Department of Agriculture’s (USDA’s) Automated Multiple-Pass Method. All foods reported in the recall were coded using the USDA’s Food and Nutrient Database for Dietary Studies (FNDDS), using the FNDDS 2015–2016 and FNDDS 2017–2018, respectively, to calculate nutrient and energy intake. 

The HEI-2015 was used to determine DQ using the population-based scoring algorithm. The HEI-2015 provides an assessment of the overall DQ and a total of 13 dietary components, which includes nine adequacy foods: total vegetables (canned, fresh), greens and beans, total fruits (juices, canned), whole fruits, total protein foods, seafood and plant proteins (each scored 0 in the lowest consumption and scored 5 per 1000 kcals in the highest consumption), whole grains, dairy, and fatty acids (each scored 0 in the lowest consumption and 10 in the highest per 1000 kcals). The fatty acids are presented as a ratio of polyunsaturated + monounsaturated fats/saturated fats, added sugars, and saturated fats as a percentage of energy. There are four moderation foods: sodium, added sugars, refined grains, and saturated fats (each scored 10 in the lowest consumption and 0 in the highest per 1000 kcals) [21,22,23]. The total HEI-2015 scores range from 0 to 100, in which a score of 50 suggests a diet of “poor” quality, scores from 50 to 80 suggest that the diet “needs improvement”, and a score 80 indicates a “good diet” and adheres closest to the Dietary Guidelines for Americans (DGAs) [17,21]. 

### 2.4. Data Analysis Plan

Descriptive statistics had been obtained for the study variables. Mean and standard deviations were reported for the continuous variables (eGFR, hs-CRP, DQ, age), and frequency and weighted percent were reported for the categorical and dichotomous variables (race/ethnicity, hypertension, diabetes mellitus, cardiovascular disease, kidney disease awareness). Four-year sample weights were calculated for the combined survey cycles using a known probability of sampling for each individual and NHANES complex survey design structure according to the NHANES analytic guidelines [24]. 

The Pearson chi-squared (Χ^2^) test was conducted to compare differences in categorical and dichotomous variables for participants with an eGFR < 60 or ≥90 mL/min/1.73 m^2^. Independent t-tests were used to compare differences in continuous variables for participants with an eGFR < 60 or ≥90 mL/min/1.73 m^2^. The assumptions of linear regression were assessed using histograms, normal Q–Q plots, and residuals. The key independent variables (hs-CRP and DQ) and the covariate, age, followed a normal distribution. Beta estimates (B) and their 95% confidence intervals (CI) were reported for each independent variable. Standard errors were obtained by Taylor series linearization. All analyses were performed in SAS version 9.4 [25]. A *p*-value < 0.05 was used to determine statistical significance.

## 3. Results

### 3.1. Descriptive Statistics of Population

The final analytic sample consisted of 6230 participants that met the inclusion criteria. Of these, 52.8% were female, 32.3% self-reported as non-Hispanic White, with a mean age of 46 years. A higher portion of participants with an eGFR < 60 were older, indicated that they had diabetes, hypertension, cardiovascular disease, and were aware that they had kidney disease compared to participants with an eGFR ≥90. Participants with an eGFR < 60 had higher amounts of the hs-CRP compared to participants with an eGFR ≥ 90 (*p* < 0.01). No differences were observed with the overall DQ scores (*p* > 0.05) based on the eGFRs (Table 1).

### 3.2. Multiple Linear Regression Analysis: Interaction Effect of DQ and hs-CRP on eGFR

In Model 1, which was unadjusted for correlates, the multivariable regression revealed that there was a significant negative interaction effect of DQ and hs-CRP on the eGFR regardless of the baseline eGFR. In Model 2, which was unadjusted for correlates, for participants with an eGFR <60, there was a statistically significant negative relationship between the hs-CRP and eGFR (β = −0.2, *p* < 0.01) and a statistically positive relationship between the DQ and eGFR (β = 0.1, *p* = 0.04). For participants with an eGFR ≥ 90, there was a statistically significant negative relationship with the hs-CRP and DQ on the eGFR (*p* < 0.001). Model 3, which adjusted for all potential correlates, demonstrated that age, kidney disease awareness, and hypertension were influencing factors for participants with an eGFR < 60. For participants with an eGFR ≥ 90, when including the covariates, there was no relationship effect of their hs-CRP and DQ on the eGFR. The covariates that influenced the eGFR were gender, age, and race/ethnicity. In Model 4, the covariates that continued to have an influence on this relationship were age, hypertension, and kidney disease awareness for participants with an eGFR < 60. Age had an influencing effect on DQ with an eGFR ≥ 90 (Table 2).

### 3.3. Multiple Linear Regression Analysis: Relationship between Dietary Components and eGFR

**Figure 1:**
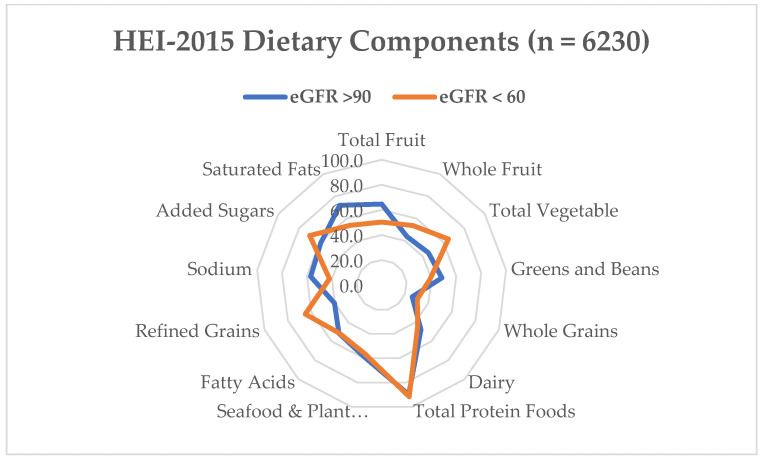
Mean Healthy Eating Index-2015 component scores as a percentage of the total possible component score for adults with eGFR < 60 (*n* = 687) and eGFR > 60 (*n* = 5543). The population ratio method was used to calculate the mean intake of each group. Dairy, total protein foods and seafood and plant proteins include alternative dairy and protein products such as soy. The numbers represent the percentage consumed from 0 to 100.

For participants with an eGFR < 60 and ≥90, the dietary components that had the highest median score were the total protein (5.0), refined grains (7.4 and 6.1, respectively) and added sugars (7.9 and 8.3, respectively). The dietary components with the lowest median scores were the greens and beans (0.4 and 1.5, respectively), whole grains (1.9 and 1.2, respectively), and total fruit (2.3 and 1.9, respectively) (Figure 1). For this analysis, the model was not adjusted for correlates. There was no significant relationship between dietary components and an eGFR at <60. For participants with an eGFR ≥ 90, the dietary components that had a statistically significant negative influence on the eGFR were the whole fruit, whole grain, and refined grains with dairy and saturated fats having a statistically significant positive influence on the eGFR (*p* < 0.05) (Table 3).

## 4. Discussion

The current study aimed to investigate the interaction effect of the hs-CRP and DQ, relationship between dietary components, and demographics and health conditions on the eGFR using the 2015–2016 and 2017–2018 NHANES data. A few of the hypotheses were proven correct as the results demonstrated that regardless of the baseline eGFR, there was a significant interaction effect of the hs-CRP and DQ on eGFR, indicating that elevated inflammation and low DQ led to a lower eGFR. For at least those participants with an eGFR < 60, the study also found that the covariates of age, hypertension, and kidney disease awareness had a negative influence on the eGFR. However, no specific dietary components or other demographics and health conditions had an impact on the eGFR. 

The median overall DQ was 52.8 out of 100 for participants with an eGFR of <60 and 53.3 out of 100 for participants with an eGFR of ≥90, which indicates a need for improvement [17,26]. Participants had higher scores in the refined grains and total proteins and lower scores in the whole grains, seafood and plant proteins, and total fruit. The interactive effect between the overall DQ and hs-CRP may have been related to these specific dietary component scores, thus impacting other nutrients like the low fiber intake. As observed in various studies, decreased fiber intake was significantly associated with an elevated hs-CRP ≥ 3 mg/L [27,28]. These findings show that higher systemic inflammation may be a significant biological process linking a lower-quality diet to negative health outcomes including eGFR decline. It is hypothesized that plant-based foods, which have low acid loads, effect inflammation modulation [27,29] by suppressing free radical production [30,31,32,33], which in effect prevents metabolic acidosis and inflammation. Moreover, seafood, which contains high amounts of omega-3 polyunsaturated fatty acids (PUFAs), has been shown to control the immune response by preventing pro-inflammatory pathways from being activated and lowering cytokine release [34]. In line with the current study’s findings, other studies have shown that certain foods have a relationship with the hs-CRP to reduce the eGFR [35,36,37]. A cross-sectional study of 1989 adults were conducted to examine the relationships between DQ, as measured by HEI-2015, and a range of chronic low-grade inflammatory biomarkers, including the hs-CRP. According to the findings, DQ was negatively related with the hs-CRP (*p* = 0.02). The study showed that consuming a high-quality diet was particularly important for controlling inflammation [36].

Examining the independent relationship between DQ and the eGFR, at least for participants with an eGFR < 60, results were consistent with recent evidence linking a Western-style diet pattern to CKD progression [14]. A retrospective study using data from NHANES III that included 1486 adults with eGFRs of ≥15 and <60 mL/min/1.73 m^2^ showed that participants who consumed meat-based diets with high acid loads, like meat, cheese, sugar, and processed foods, had a higher risk of progressing to the final stage of kidney disease than those who consumed less acidic foods, like fruit, vegetables, legumes, and potatoes. The results of their study showed that acid-inducing diets caused the eGFR to decrease over time and increased acid excretion, which may cause kidney damage in adults with moderate and advanced CKD [38]. Consuming more animal-based proteins activates glucagon secretion in contrast to plant-based proteins. This causes the vasodilation of the afferent arteriole and glomerular hyperfiltration, and increases insulin resistance, leading to vascular injury and resulting in albuminuria, with a decline in kidney function [39]. Furthermore, during the metabolism of animal-based proteins, acidic byproducts are produced, which increases plasma amino acid levels, CRP levels, and increases uremic toxins. On the other hand, as plant-based proteins are rich in fiber, when metabolizing these proteins, there is a reduction in the production of uremic toxins. This is mainly due to the role that fibers play in reducing protein fermentation [40]. Similarly, dietary fiber in fruits, vegetables, legumes, and whole grains protects kidney hemodynamics by reducing the production of the acid load and improving the intestinal microbiota composition. Legumes, nuts, and seeds have been recommended as high-quality sources of plant protein due to their higher proportion of essential amino acids compared to other plant foods [41]; nonetheless, their overall consumption was found to be low in this current study.

As a result of poor DQ, nutrient overload may result in a systemic metabolic dysfunction, which can cause kidney dysfunction [42,43]. In particular, the combination of high levels of added sugar, salt, and saturated fat may impair kidney vascularization. To calculate the overall DQ, these specific dietary components are reverse scored, in which a higher score indicates less consumption whereas a lower score indicates a higher consumption. At least for added sugars in this study, the median score was high, >7.0 out of 10, indicating less consumption of this nutrient. The intake of added sugar, particularly fructose, which is primarily consumed in the United States in the form of sugar-sweetened beverages and artificial sweeteners, contributes to insulin resistance, raises blood uric acid, and has impacts on endothelial function [44,45]. This results in a decline in kidney function. As limited information is provided in NHANES about the serving amount consumed with certain foods that may contain added sugars, it is challenging to determine if participants were consuming elevated amounts of fructose which may have contributed to low eGFRs. In this study, the median scores of sodium and saturated fat were low, <4 and <6, respectively. An overconsumption of saturated fats can result in oxidative stress in cells and mitochondrial dysfunction, both of which are essential factors in the process of kidney damage [46]. Consequently, higher dietary scores and better dietary adherence could slow the progression of CKD by reducing uremic toxins, oxidative stress, and inflammation.

The following may provide an explanation for the inverse relationship between the hs-CRP and the eGFR. In this study, adults with elevated hs-CRP levels (*n* = 3141) accounted for 50.4% of the study sample. Inflammation has been linked to both early endothelial dysfunction and advanced atherosclerosis due to a dysfunctional glomerular endothelium and/or impaired autoregulation of glomerular pressure [47,48]. Specifically, the CRP stimulates the production of proinflammatory cytokines, which induces mesangial cell proliferation, matrix overproduction, and an increased permeability of the vascular system, all of which alters membrane permeability and leads to albuminuria [49]. The findings from this current study are consistent with other studies [50,51]. The Chronic Renal Insufficiency Cohort (CRIC) examined the effects of kidney function on inflammation, including the hs-CRP, among adults (*n* = 3939) with a eGFR < 60 mL/min/1.73 m. Results demonstrated that inflammatory biomarkers including the hs-CRP were negatively associated with kidney function (cystatin C and the eGFR) [51]. The authors of this study mentioned that in the early stages of kidney disease, cytokine-mediated inflammation is already present, especially in the aging population. This was further demonstrated in their results in which moderately elevated albuminuria was related to adverse risk factors such as age, high blood pressure, and elevated inflammatory markers in participants with or without diabetes.

The current study demonstrated that for participants with an eGFR of <60, hypertension had a negative influence on eGFR within this model, which may have been due to these pathophysiologic states that are intimately related. Persistent hypertension can deteriorate kidney function, while CKD can worsen blood pressure regulation. The etiology of hypertension in CKD is complicated and the result of a number of factors, including decreased glomerular mass, decreased sodium excretion and an extracellular volume expansion, an overactive sympathetic nervous system that increases peripheral vasoconstriction, an activation of hormones including the renin-angiotensin-aldosterone system (RAAS), and endothelial dysfunction [52]. Several studies have reported the relationship between hypertension and eGFR decline among adults with CKD [53,54]. Possibly, in this study, the low total fruit and vegetable consumption may have led to this result. Vegetables are the main source of dietary nitrate and nutrients with low bioavailability, such as phosphorus and protein, that influence kidney function in compromised individuals. Nitrates in the diet have the potential to lower systolic blood pressure which protects kidney function. High blood pressure raises glomerular pressure, which in turn stretches the glomerular capillary wall, damages the endothelium, and boosts protein filtration through the glomeruli [55]. Thus, consuming more plant-based foods would contribute to the reduction in blood pressure and progression of CKD.

There was a negative influence of kidney disease awareness on the eGFR. In this study, 72.9% of participants with an eGFR < 60 were not aware that they had kidney disease even though the mean eGFR fell into CKD stage 3. This is in alignment with the Centers for Disease Control (CDC) findings, in which 9 out of 10 adults did not know that they have this disease [1]. A cross-sectional study was conducted to determine the kidney disease awareness through the risk of progression to kidney failure of adults with eGFRs of >15 to <60 mL/min/1.73 m^2^ (n = 3713) using NHANES data from 1999 to 2016. Results showed that only about half of the participants who had a high risk for kidney failure were aware of their disease and this remained stagnant throughout the years. The authors contributed this unawareness to the limited discussion during primary care visits and the adults’ lack of knowledge about CKD in general [56]. The lack of awareness about CKD in this study may have also contributed to the DQ and low consumption of certain dietary components. Therefore, more attention should be placed on CKD awareness campaigns and discussing proper dietary patterns to reduce and prevent the development of CKD.

### Limitations

The study has several limitations that should be taken into consideration when interpreting the results. First, due to the cross-sectional design of the study, it is not possible to establish causal relationships between the variables under investigation. Second, the study is susceptible to both selection and response biases (self-reported conditions, race, age, gender), which limits the generalizability of these findings. In addition, the NHANES dataset has some limitations regarding missing data and limited follow-up, which may restrict its usefulness for studying longitudinal health outcomes. Another limitation is that the hs-CRP and the determination of the eGFR were based on a single day’s measurement, which could result in some overestimation. However, any potential bias resulting from variations in the eGFR would most likely conservatively bias the regression estimates. Furthermore, the number of participants with advanced CKD was small, and further studies should examine whether the observed associations hold among individuals with more advanced kidney disease.

## 5. Conclusions

Overall, this cross-sectional study provides new insights into the complex relationship between hs-CRP, DQ, and eGFR among the US adult population. Findings demonstrated a negative interaction effect of inflammation and overall DQ on eGFR. The overall DQ was considered to be in need of improvement with a lower consumption of whole grains, greens and beans, and whole fruit, regardless of the eGFR. The findings of this study highlight the importance of overall dietary habits and the presence of inflammation on the eGFR. Further research is needed to better understand the mechanisms underlying the observed relationships and to develop effective interventions to improve our understanding of kidney disease prevention and progression through preventing and treating abnormal eGFRs in the United States population.

## Figures and Tables

**Table 1:** Weighted characteristics of the NHANES participants based on eGFRs (≥90 or <60). (2015–2018) (*N* = 6230).

Characteristics	eGFR ≥ 90	eGFR < 60	*p*-Value *
	*N* = 5543	*N* = 687	
Age, mean ± S.E., y	43.5 ± 15.1	70.2 ± 11.1	<0.001 *
Sex (female), %	2936 (53.0%)	352 (51.2%)	0.2
Race/ethnicity, %			<0.001 *
Mexican American	1106 (20.0%)	67 (9.8%)	
Other Hispanic	724 (13.1%)	45 (6.6%)	
Non-Hispanic White	1707 (30.8%)	307 (44.7%)	
Non-Hispanic Black	935 (16.9%)	228 (33.2%)	
Non-Hispanic Asian	1071 (19.3%)	40 (5.8%)	
Other			
Health conditions			
Diabetes (yes), %	759 (13.7%)	262 (38.1%)	<0.01 *
Hypertension (yes), %	1513 (27.3%)	522 (76.0%)	<0.01 *
Cardiovascular disease (yes), %	325 (5.9%)	259 (37.7%)	<0.01 *
Kidney disease awareness (yes), %	77 (1.4%)	186 (27.1%)	<0.01 *
hs-CRP mg/L, mean ± S.E.	3.9 ± 7.2	5.7 ± 10.2	<0.01 *
DQ, median (25th, 75th percentile)	52.8 (12.7, 62.0)	53.3 (43.8, 63.1)	0.2
eGFR mL/min/1.73 m^2^, mean ± S.E.	108.4 ± 11.5	46.4 ± 11.8	<0.01 *

All percentages and means ± S.E. were adjusted for survey weights. Comparison between eGFRs for categorical data, *p*-value was estimated using chi-square for categorical data and t-test for continuous data. *p*-value *: *p* < 0.05 is considered statistically significant. eGFR: estimated glomerular filtration rate, hs-CRP: high-sensitivity C-reactive protein, DQ: diet duality.

**Table 2:** Multivariate regression with interaction effect of hs-CRP and DQ on eGFR (*N* = 6230).

Model	Parameter	eGFR ≥ 90	eGFR < 60
		β (95% CI) *	β (95% CI) *
1	Interaction: DQ×hs-CRP	−0.0 (−0.0–0.0) *	−0.004 (46.5–48.5) *
2	hs-CRP	−0.1 (−0.1–−0.0) *	−0.2 (−0.3–−0.1) *
	DQ	−0.1 (−0.1–−0.1) *	0.1 (0.0–0.1) *
3	hs-CRP	0.0 (−0.0–0.0)	−0.1 (−0.2–−0.0) *
	DQ	0.0 (−0.0–0.0)	0.1 (0.0–0.1)*
	Gender	2.1 (1.7–2.6) *	−0.5 (−2.2–1.3)
	Age	−0.5 (−0.5–−0.5) *	−0.1 (−0.7–−0.00) *
	Mexican American	4.6 (3.9–5.2) *	0.01 (−4.4–4.5)
	Other Hispanic	2.5 (1.7–3.2) *	0.8 (−4.0–5.7)
	Non-Hispanic White	−2.2 (−2.7–−1.7) *	2.7 (−1.1–6.5)
	Non-Hispanic Black	−2.3 (−3.0–−1.7) *	1.3 (−2.6–5.1)
	Other	Ref.	
	Hypertension	−0.1 (−0.7–0.5)	−3.9 (−2.5–1.1) *
	Diabetes	1.7 (1.0–2.4) *	−0.8 (−2.6–1.0)
	Cardiovascular Disease	0.0 (−0.9–1.0)	−0.7 (−2.5–1.1)
	Kidney awareness	0.0 (−1.9–1.9)	−8.9 (−10.9–−7.0) *
4	hs-CRP	0.0 (−0.0–0.1)	−0.1 (−0.2–−0.5) *
	DQ	0.03 (0.0–0.1) *	0.1 (0.0–0.1) *
	Age	−0.5 (−0.5–−0.5) *	−0.1 (−0.2–−0.0) *
	Hypertension	−0.5 (−1.0–0.1)	−4.3 (−6.1–−2.4) *
	Kidney awareness	0.7 (−1.3–2.6)	−9.2 (−11.1–−7.4) *

β: Beta, CI: Confidence Interval, DQ: refers to diet quality, hs-CRP: high-sensitivity C-reactive protein, eGFR: estimated glomerular filtration rate, HTN: hypertension, DM: diabetes mellitus, CVD: cardiovascular disease, * *p*-value: *p* < 0.05 is considered statistically significant, Ref.: reference.

**Table 3:** Multivariable results of the independent effect of DQ and the individual dietary components on eGFR (*N* = 6230).

Dietary Components	eGFR ≥ 90		eGFR < 60	
	Median of DQ Scores (25th, 75th)	β (95% CI) *	Median of DQ Scores (25th, 75th)	β (95% CI)
Total vegetables	3.3 (2.0, 5.0)	−0.2 (−0.4–0.1)	3.3 (1.9, 5.0)	0.1 (−0.6–0.8)
Greens and Beans	1.5 (0.0, 5.0)	0.2 (−0.0–0.3)	0.4 (0.0, 5.0)	0.3 (−0.1–0.8)
Total Fruit	1.9 (0.1, 4.8)	0.2 (−0.1–0.5)	2.3 (0.4, 5.0)	−0.1 (−0.9–0.8)
Whole Fruit	2.2 (0.0, 5.0)	−0.5 (−0.7–−0.2) *	3.0 (0.0, 5.0)	0.4 (−0.3–1.1)
Whole Grain	1.2 (0.0, 4.3)	−0.1 (−0.2–−0.2) *	1.9 (0.0, 5.3)	−0.2 (−0.5–0.1)
Dairy	4.4 (2.3, 7.0)	0.2 (0.1–0.3) *	3.9 (1.9, 6.5)	0.2 (−0.1–0.6)
Total Protein	5.0 (4.5, 5.0)	−0.1 (−0.5–0.2)	5.0 (4.7, 5.0)	0.2 (−1.0–1.4)
Seafood and Plant Proteins	3.8 (0.2, 5.0)	−0.0 (−0.2–0.2)	3.5 (0.0, 5.0)	0.1 (−0.3–0.6)
Fatty Acids	4.9 (2.2, 8.3)	−0.1 (−0.2–0.1)	4.8 (2.4, 8.2)	0.2 (−0.2–0.6)
Sodium	3.8 (0.7, 6.5)	−0.1 (−0.2–0.0)	4.1 (1.2, 6.7)	0.2 (−0.1–0.6)
Refined Grains	6.1 (2.7, 9.4)	−0.3 (−0.4–−0.2) *	7.4 (4.2, 10.0)	0.2 (−0.2–0.5)
Saturated Fats	6.2 (3.4, 9.0)	0.2 (0.1–0.3) *	5.5 (2.6, 8.4)	−0.1 (−0.5–0.3)
Added Sugars	8.3 (5.4, 10.0)	−0.1 (−0.2–0.0)	7.9 (4.9, 10.0)	0.0 (−0.3–0.4)

HEI: Healthy Eating Index, N: sample size, DQ: Diet Quality, eGFR: estimated Glomerular Filtration Rate, B^1^: DQ components estimate (B), *: indicates *p* < 0.05 contributing to statistical significance.

## Data Availability

Data are available on the NHANES website, and the analytic codes will be made available pending email request to the corresponding author.

## Supplementary Materials

The following supporting information can be downloaded at: https://www.mdpi.com/article/10.3390/nu16040528/s1, Figure S1: Participant Flow Chart.
