# Supplementary figures and images for: Elevated Inflammation and Poor Diet Quality Associated with Lower eGFR in United States Adults: An NHANES 2015–2018 Analysis
Source: Nutrients. 2024 Feb 14;16(4):528. doi: 10.3390/nu16040528 (PMC10891552; doi:10.3390/nu16040528)

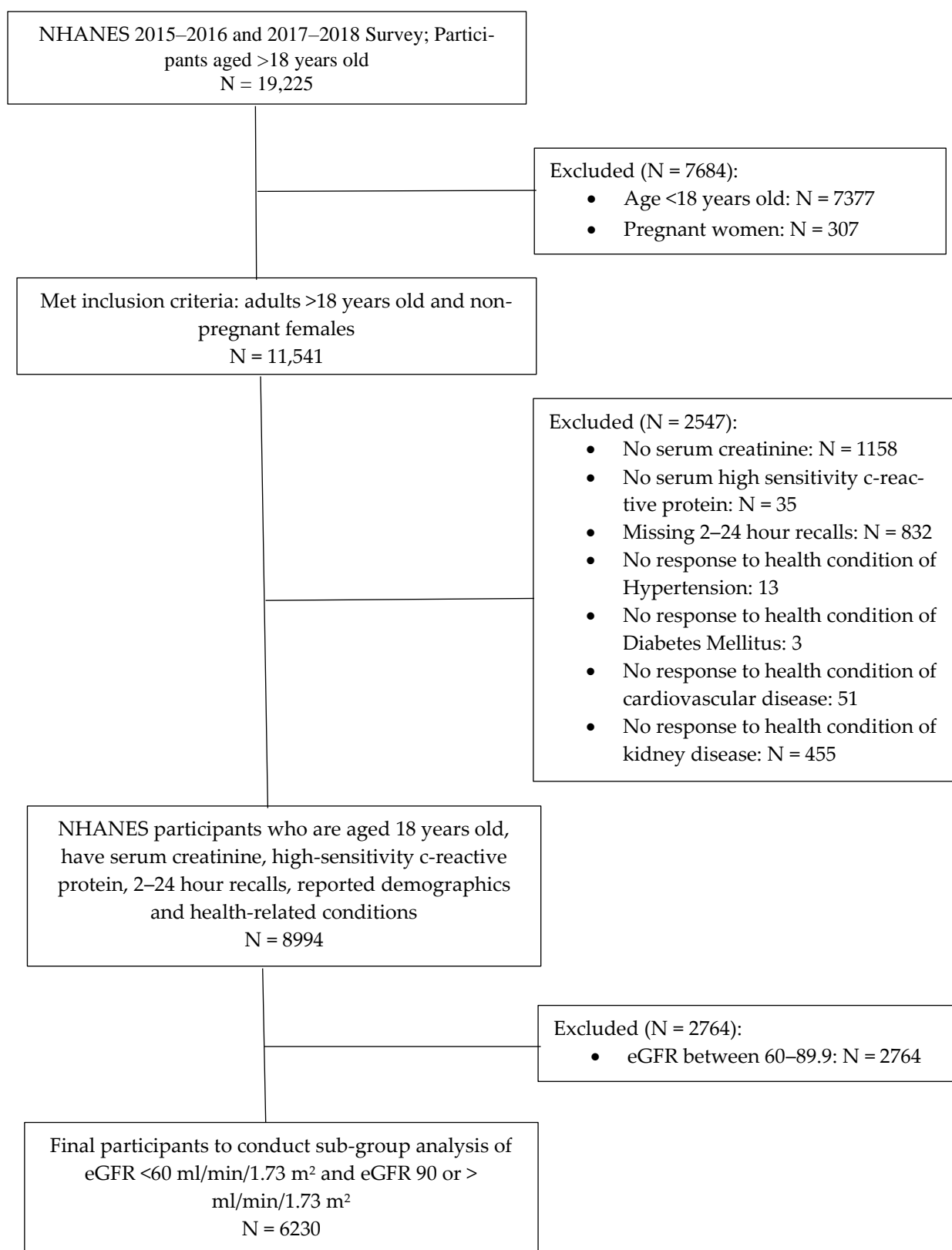

**Figure S1.** Participant Flow Chart

Supplement: Supplementary file 1 [file nutrients-16-00528-s001.zip › nutrients-2802313-supplementary.pdf]
